# Intraocular Pressure Changes in Non-Surgical Eyes of Patients Admitted for Glaucoma Surgery

**DOI:** 10.3390/jcm13154511

**Published:** 2024-08-01

**Authors:** Suguru Kubota, Soichiro Shimomine, Yoichi Kadoh, Masaki Tanito

**Affiliations:** Department of Ophthalmology, Shimane University Faculty of Medicine, Izumo 693-8501, Japanykadoh@med.shimane-u.ac.jp (Y.K.)

**Keywords:** glaucoma medication, medication adherence, non-surgical eyes, older age, cognitive function

## Abstract

(1) **Background**: In glaucoma patients, it is observed that intraocular pressure (IOP) in non-surgical eyes decreases during hospitalization, but detailed data have not been reported. This study aimed to examine changes in IOP in the non-surgical eyes of patients admitted for glaucoma surgery. (2) **Methods**: This retrospective study included 487 patients (254 males, 233 females). Statistical analysis was performed separately for groups that were and were not under medication treatment. (3) **Results**: In non-surgical eyes, the difference in IOP between admission and discharge was −1.6 ± 4.8 mmHg (95% confidence interval (CI), −2.1 to −1.2; *p* < 0.0001) with a significant decrease in the medication group (n = 414), while it was −0.6 ± 4.8 mmHg with no significant change observed (95% confidence interval (CI), −1.7 to 0.57; *p* = 0.33) in the non-medication group (n = 73). Multiple regression analysis of the medication group showed that IOP at admission (*p* < 0.0001) and older age (*p* = 0.03) were associated with the reduction or the rate of reduction in IOP. (4) **Conclusions**: IOP in the non-surgical eyes of patients admitted for glaucoma surgery with medication decreased during hospitalization. The reduction was more pronounced in individuals with higher IOP at admission and in older patients. It is possible that improved medication adherence contributes to the lowering of IOP.

## 1. Introduction

Glaucoma is a group of diseases that cause irreversible vision loss and are characterized by the progressive disorder of retinal ganglion cells [1]. Optic nerve atrophy due to glaucoma is termed glaucomatous optic neuropathy. Glaucoma is broadly divided into open-angle and angle-closure glaucoma. The most common form is primary open-angle glaucoma, but primary angle-closure disease and exfoliation glaucoma are also major types in the elderly [2]. It is estimated that the global prevalence of glaucoma among people aged 40 to 80 years is 3.54% and that the number of glaucoma patients worldwide will increase to 111.8 million by 2040 [3]. Previous large clinical trials have shown that intraocular pressure (IOP) is the only modifiable risk factor [1]. Therefore, lowering IOP is important in the treatment of glaucoma, and IOP-lowering treatments include glaucoma medications, surgery, and laser treatment [4]. Medication is currently the most common initial intervention to lower IOP. Incisional surgery is generally an option considered in patients using glaucoma medications and can be indicated if IOP is insufficiently lowered [1,5].

A phenomenon in which IOP in glaucoma patients decreases during hospitalization has been observed [6,7,8]. This decrease was more pronounced among treated patients [8]. Therefore, it seems possible that hospitalization has the effect of lowering IOP in glaucoma patients, but the mechanism is not fully understood. Clinical data on changes in IOP in non-surgical eyes during hospitalization for glaucoma surgery have not been reported because the above papers reporting a reduction in IOP during hospitalization did not target hospitalized patients admitted for glaucoma surgery. One of the factors related to an IOP decrease during hospitalization is considered adherence. Older age and lower cognitive function are risks for failed eye-drop instillation [9]. Hospitalized patients are administered eye drops by nurses. For patients with poor adherence to eye drops, such as older individuals or those with decreased cognitive function, improvement in adherence during hospitalization may lower IOP.

The purpose of this study was to examine changes in IOP in the non-surgical eyes of patients admitted for glaucoma surgery in a single eye. We also investigated parameters that influence these changes to clarify the relationship with factors such as age and cognitive function.

## 2. Subjects and Methods

### 2.1. Study Design and Subjects

We conducted a retrospective study based on medical record information. This study followed the principles outlined in the Declaration of Helsinki and the Ethical Guidelines for Medical and Health Research Involving Human Subjects in Japan. The Institutional Review Board (IRB) of Shimane University Hospital reviewed and approved the research (Approval No. 20220616-1, issued on 3 April 2023). The IRB approval did not necessitate written informed consent from each patient for publication. Instead, the study protocol was made available at the study institutions, allowing participants to opt out if they wished to do so. Subjects were recruited consecutively at the Department of Ophthalmology, Shimane University Hospital, from March 2020 to April 2023. All subjects who met the inclusion criteria were enrolled in the study. These criteria encompassed patients who were admitted to our hospital and underwent glaucoma surgery by the same surgeon (M.T.), patients whose IOP was measured at admission and discharge in both eyes, and patients who underwent a cognitive function test using the Mini-Cog assessment. Cases with unstable IOP measurements (phthisis bulbi, remarkable corneal irregularities, etc.) were excluded. However, pterygium and previous refractive surgery were not excluded. In the case of multiple hospitalizations, the hospitalization closest to the most recent cognitive function evaluation date was considered.

### 2.2. Measurements

We collected data on age at the cognitive function test, sex, cognitive function, length of hospital stay, glaucoma type, best-corrected visual acuity (BCVA), IOP at admission and discharge, medication score (MS) at admission and discharge, spherical equivalent refractive error (SERE), and mean deviation (MD) from medical records. BCVA, IOP, MS, SERE, and MD were measured in surgical and non-surgical eyes. The MS was defined as the number of prescriptions for glaucoma eye drops and oral medications, and eye drops were counted as two if they were a fixed dose. Cognitive function was estimated with the Mini-Cog test, a composite of three-item word memory and clock drawing [10]. This test has a score ranging from 0 (poor) to 5 (good), with a score of 2 or worse indicating suspected dementia [11]. The decimal BCVA was converted into the logarithm of the minimum angle of resolution (LogMAR). IOP at admission and discharge was measured by the iCare rebound tonometer TA01i (RBT) at a consultation between 7:00 and 10:00 a.m. If IOP at admission was not recorded, the most recent RBT-IOP value measured at the outpatient clinic of our hospital before admission was used as a substitute. Outpatient IOP was measured by the RBT between 8:30 a.m. and 12:00 noon. In our hospital, IOP was measured by the RBT and Goldmann applanation tonometer (GAT) in the outpatient clinic and by the RBT in patients immediately after surgery. Therefore, RBT measurements for which pre- and post-operative data were available were used as the IOP values in the present study. SERE was measured by autorefractometry (TonoRef III, Nidek, Gamagori, Japan).

### 2.3. Statistical Analysis

The data were statistically analyzed separately for those who were on glaucoma medication for non-surgical eyes before surgery and those who were not. The data are presented as mean ± standard deviation (SD) with 95% confidence interval (CI) ranges for continuous parameters and as numbers and percentages for categorical parameters. The potential association between patients on medication and those not on medication was evaluated using an unpaired *t*-test for continuous parameters and Fisher’s exact probability test for categorical parameters. The difference between IOP values at admission and at discharge (ΔIOP, IOP at discharge minus IOP at admission) and the difference between medication scores at admission and discharge (ΔMS, medication score at discharge minus medication score at admission) were evaluated through the paired *t*-test. IOP at admission was classified into 4 groups based on its magnitude, and the change in each IOP was assessed through one-way ANOVA. Additionally, potential associations between ΔIOP, ΔMS, %ΔIOP (ΔIOP divided by IOP at admission), or %ΔMS (ΔMS divided by MS at admission) and various parameters were explored through multiple regression analysis. All statistical analyses were conducted using JMP Pro statistical software version 16.1.0 (SAS Institute, Inc., Cary, NC, USA). A *p* value of less than 0.05 was considered statistically significant.

## 3. Results

The demographic data of the subjects are presented in Table 1. The number of subjects on glaucoma medication before glaucoma surgery was 414, and that without was 73. There were no significant differences in age, sex, or Mini-Cog score between the medication group and the non-medication group. The length of hospital stay was significantly longer in the medication group. The glaucoma type was classified into primary open-angle glaucoma (PG), exfoliation glaucoma (EG), and other (including patients without glaucoma). PG (61.6%) was the most common in the medication group, but PG (41.1%) and other (43.8%) were equally common in the non-medication group.

Table 2 shows IOP at admission and discharge. IOP in surgical eyes decreased significantly during hospitalization in the medication and non-medication groups. In non-surgical eyes, IOP at admission was 14.8 ± 5.5 mmHg, and IOP at discharge was 13.2 ± 5.8 mmHg, with a significant decrease (ΔIOP = −1.6 ± 4.8, *p* < 0.0001) in the medication group. On the other hand, in the non-medication group, IOP at admission was 13.9 ± 6.0 mmHg, and IOP at discharge was 13.3 ± 6.9 mmHg, with no significant change observed (ΔIOP = −0.6 ± 4.8, *p* = 0.33). There was no significant difference in IOP at admission in non-surgical eyes at admission between the medication and non-medication groups.

Table 3 presents the MS at admission and discharge. In non-surgical eyes, there was a significant difference in the MS between the medication and non-medication groups. In the medication group, the MS at admission was 3.1 ± 1.2, and the MS at discharge was 2.3 ± 1.3 (ΔMS = −0.9 ± 1.3, *p* < 0.0001). In the non-medication group, the MS at admission was 0 ± 0, and the MS at discharge was 0.5 ± 1.0 (ΔMS = 0.5 ± 1.0, *p* < 0.0001).

**Figure 1:**
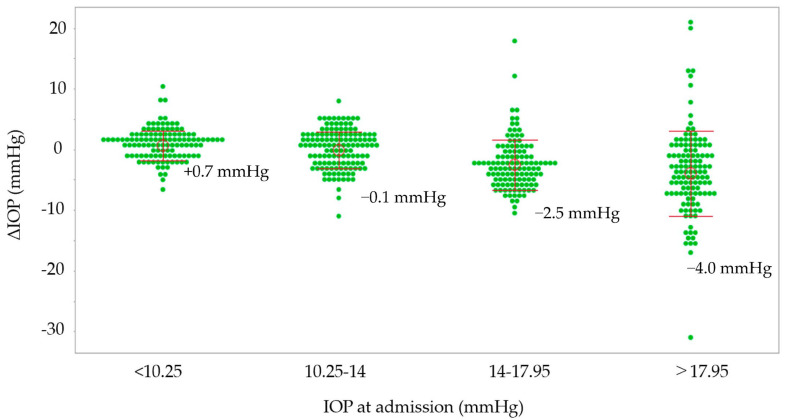
The relationship between IOP at admission and IOP changes and ΔIOP. The groups are stratified by the quartile values of IOP at admission. IOP, intraocular pressure; ΔIOP, IOP at discharge minus IOP at admission.

Possible associations between ΔIOP and various parameters are shown in Table 4. In the medication group, high IOP at admission (*p* < 0.0001) was associated with a reduction in IOP in the non-surgical eyes, and a long hospital stay (*p* = 0.029) was associated with an increase in IOP in the non-surgical eyes. In the non-medication group, other glaucoma types (*p* = 0.044) and high IOP at admission (*p* < 0.0001) were associated with a reduction in IOP in the non-surgical eyes. Possible associations between %ΔIOP and various parameters are shown in Table 5. In the medication group, high IOP at admission (*p* < 0.0001) and older age (*p* = 0.03) were associated with a reduction in IOP in non-surgical eyes, and a long hospital stay (*p* = 0.01) was associated with an increase in IOP in non-surgical eyes. In the non-medication group, other glaucoma types (*p* = 0.037), high IOP at admission (*p* < 0.0001), and a long hospital stay (0.045) were associated with a reduction in IOP in non-surgical eyes. Both groups were classified into four classes according to the quartile value of IOP at admission, and the relationship between IOP at admission and IOP changes is shown in Figure 1. In the IOP groups with IOP at admission exceeding 17.95 mmHg and 14–17.95 mmHg, IOP decreased by 4.4 mmHg and 2.5 mmHg, respectively, during hospitalization. In the IOP group with 10.25–14 mmHg, the IOP reduction was 0.1 mmHg, and in the IOP group with lower than 10.25 mmHg, IOP increased by 0.7 mmHg.

Possible associations between ΔMS and various parameters are presented in Table 6. In the medication group, older age (*p* = 0.0026), a high MS at admission (*p* < 0.0001), and the MD (*p* = 0.005) were associated with a reduction in IOP in non-surgical eyes. In the non-medication group, there were no factors associated with IOP changes in non-surgical eyes. In Table 7, possible associations between %ΔMS and various parameters are presented. In the medication group, older age (*p* = 0.001) and high MS at admission (*p* < 0.0001) were associated with a reduction in IOP in non-surgical eyes.

## 4. Discussion

To the best of our knowledge, the current study is the first to present data on IOP changes in the non-surgical eyes of patients admitted for glaucoma surgery. Previous studies [6,7,8] have shown that hospitalization lowered IOP in glaucoma patients, but these subjects were only hospitalized and did not undergo glaucoma surgery. Hyams et al. reported that IOP was lower during hospitalization in 13 subjects with open-angle glaucoma or ocular hypertension receiving antiglaucoma therapy or not [6]. Kashiwagi et al. measured inpatient IOP and ambulatory IOP in 52 normal-tension glaucoma patients and showed that inpatient IOP was significantly lower than ambulatory IOP [7]. Haufschild’s group reported that IOP decreased significantly in 26 high-tension and 13 normal-tension glaucoma patients under IOP-lowering treatment and 28 normal-tension glaucoma patients without IOP-lowering treatment. By examining changes in IOP in hospitalized patients undergoing glaucoma surgery, we were able to obtain more realistic clinical data. In addition, the number of subjects was also larger. In this study, IOP in non-surgical eyes decreased only in the medication group but not in the non-medication group (Table 2). IOP in the surgical eyes decreased significantly in both the medication group and the non-medication group. In the study by Haufschild’s group, IOP decreased more in patients who were treated with medication than in patients without it [8]. In the paper, the authors discuss the influence of better medication compliance during hospitalization, mental stress, and autonomic nervous system changes as causes of IOP reduction.

While patients are in our hospital, nurses provide instructions on using eye drops or administer them. Receiving instructions on eye-drop instillation is significantly associated with a patient’s accurate eye-drop technique [12]. Hospitalization may improve medication adherence and lower IOP in non-surgical eyes. Previous discussions suggest that medication adherence may be associated with changes in IOP in the non-surgical eyes of glaucoma patients during hospitalization. The medication and non-medication groups were classified into four groups according to the IOP in non-surgical eyes at admission, and the reduction in IOP was greater in the high-IOP group (Figure 1). It is possible that, compared with lower-IOP groups, higher-IOP groups included more patients with poor medication adherence and therefore obtained a larger IOP-lowering effect by improving medication adherence through hospitalization.

Through multiple regression analysis, in the medication group, a decrease in IOP in non-surgical eyes was associated with high IOP at admission and older age (Table 4 and Table 5). As shown in Figure 1, the higher the IOP at admission, the greater the decrease in IOP. A survey conducted using a questionnaire to investigate the adherence status of glaucoma patients found that the older the patient, the better their compliance with eye drops [13]. On the other hand, according to our prior report that revealed factors associated with instillation failure among glaucoma patients with video recordings, older age is one of the factors in failed instillations [9]. In other words, although older glaucoma patients have poor eye-drop adherence because they are not good at using eye drops, hospitalization may be highly effective in improving eye-drop techniques for them. In this previous study, not only older age but also lower cognitive function, less myopic objective refractive error, and decreased foveal sensitivity were risks for failed eye-drop instillation [9]. Decreased cognitive function is expected to be associated with disorders of learning or memory function [9]. Patients with hyperopia or decreased foveal sensitivity have difficulty seeing the tip of the medication bottle and tend to fail to effectively instill the drops [14]. However, in the current study, the Mini-Cog score (≤2), SERE, and MD were not associated with IOP or %ΔIOP. It is possible that patients with these characteristics had family members or caregivers administer eye drops for them. Paradoxically, elderly people tend to have poor eye-drop technique, even if they appear healthy, so it may be necessary to consider instructing family members/carers to administer eye-drop medications if necessary.

IOP in non-surgical eyes increased as the length of hospital stay increased in the medication group and decreased in the non-medication group. Considering that hospitalization may improve eye-drop adherence, this result seems difficult to understand. However, it is possible that the number of medications was adjusted depending on the condition of the surgical eyes. For example, systemic acetazolamide is one of the options to lower IOP [15], and it is effective for both surgical and non-surgical eyes. The number of medications may be reduced in the medication group, while it may be increased in the non-medication group. It has been reported that IOP can increase in the non-surgical eye following glaucoma surgery in one eye [16]. In that report, preoperative acetazolamide use was a risk factor for increased IOP in the non-surgical eye [16]. However, in the current study, the length of hospital stay was not associated with ΔMS or %ΔMS in the medication group (Table 3), and there is no sufficient evidence to support that. Another perspective is the psychological stress from hospitalization. IOP is controlled by the autonomic nervous system, and psychological stress significantly increases IOP [17,18]. It is possible that psychological stress due to a long hospital stay was related to the increase in IOP. Compared to the sitting and standing positions, the supine position is known to increase IOP [19]. During hospitalization, patients spend more time in the supine resting position on a bed. Therefore, changes in resting position due to hospitalization might influence the relationship between the length of hospitalization and IOP.

In the non-medication group, IOP in the non-surgical eyes of patients with other glaucoma types decreased. Although it is not easy to interpret this result, one possibility is the effect of treatments other than IOP-lowering medications on the surgical eyes. Typical medications for intravitreal injections to manage retinal diseases are anti-vascular endothelial growth factor (anti-VEGF) medications and corticosteroids [20]. Anti-VEGF therapy is used for neovascular glaucoma [21], and corticosteroids are indicated for non-infectious posterior uveitis [22]. In our study, other types included these diseases.

The MS decreased significantly in the medication group and not in the non-medication group (Table 3). This effect of decreasing MS for non-surgical eyes during hospitalization may be temporary, considering that improved medication adherence during hospitalization is not permanent. However, from another perspective, if we can support patients with poor medication adherence, the number of prescribed medications may be reduced. In our previous study, older age was associated with a larger over-prescription volume [23]. These results suggest that supporting older patients may be good for the health economy.

This study also had some limitations. First, not all patients’ IOP values were measured at the exact same time. IOP is known to fluctuate throughout the day, including in glaucoma patients [24,25]. In this study, measurements were taken within the same time periods of the day [7:00–10:00 a.m. for admission (or 8:30 a.m.–12:00 noon in outpatient clinic) and 7:00–10:00 a.m. for discharge], but there is the possibility that slight differences in the time of day might have an effect. The concentration of the medication at the outpatient clinic was often at the peak value, and that at the time of admission or discharge was often at the trough value. Therefore, while the IOP difference between admission and discharge may have been underestimated, the results are not likely to be reversed. For data availability, IOP values measured by an RBT rather than a GAT were used in this study. This may be a limitation because the GAT is generally considered the gold standard for IOP measurement. However, the RBT may be more accurate than the GAT in cases where it is difficult to open the eyelid immediately after surgery due to pain or other reasons. In the present study, all patients who met the criteria were included. As a result, the study was able to include a sufficiently large number of cases and showed a significant difference in IOP before and after hospitalization in non-surgical eyes, which was the primary endpoint. However, the absence of a prior sample size calculation might be one of the limitations of the study. The medication and non-medication groups were not well balanced in terms of glaucoma type. In the future, it will be necessary to examine changes in IOP for each type.

## 5. Conclusions

This retrospective study revealed that IOP in the non-surgical eyes of patients admitted for glaucoma surgery decreased by 1.6 mmHg during hospitalization if they were under medication treatment. The reduction was more pronounced the higher the IOP at admission and the older the patient. It is possible that improved medication adherence because of hospitalization, such as medication management and eye-drop administration by medical personnel, is involved in lowering IOP in non-surgical eyes.

## Figures and Tables

**Table 1:** Demographic data.

Parameters	Glaucoma Medication (+) n = 414		Glaucoma Medication (−) n = 73		*p* Value
	N or Mean ± SD	% or 95% CI Range	N or Mean ± SD	% or 95% CI Range	
**Subjects**					
Age, years	71.6 ± 11.8	70.4, 72.7	73.7 ± 9.9	71.4, 76.0	0.15
Sex					
Male	219	52.9	35	47.9	0.45
Female	195	47.1	38	52.1	
Mini-Cog score					
≥3	387	93.5	64	87.7	0.090
≤2	27	6.5	9	12.3	
Length of hospital stay, days	4.5 ± 1.8	4.3, 4.6	3.9 ± 1.2	3.7, 4.2	0.012 *
Glaucoma type in non-surgical eyes					
PG	255	61.6	30	41.1	0.0025 *
EG	65	15.7	11	15.1	
Other	94	22.7	32	43.8	
**Non-surgical eye**					
BCVA, LogMAR	0.22 ± 0.56	0.17, 0.28	0.23 ± 0.51	0.11, 0.35	0.94
IOP at admission, mmHg	14.8 ± 5.5	14.3, 15.4	13.9 ± 6.0	12.5, 15.2	0.17
MS at admission	3.1 ± 1.2	3.0, 3.2	0 ± 0	0, 0	<0.0001 *
SERE, D	−1.9 ± 3.1	−2.2, −1.6	−1.4 ± 3.0	−2.1, −0.7	0.26
MD, dB	−7.7 ± 7.1	−8.5, −7.0	−4.5 ± 5.6	−5.9, −3.1	0.0009 *
**Surgical eye**					
BCVA, LogMAR	0.25 ± 0.43	0.21, 0.29	0.44 ± 0.67	0.28, 0.59	0.002 *
IOP at admission, mmHg	18.3 ± 8.7	17.5, 19.1	19.4 ± 9.3	17.2, 21.5	0.36
MS at admission	3.3 ± 1.2	3.2, 3.4	1.5 ± 1.6	1.2, 1.9	<0.0001 *
SERE, D	−2.1 ± 3.1	−2.4, −1.8	−1.7 ± 3.0	−2.4, −1.0	0.30
MD, dB	−11.1 ± 7.6	−11.9, −10.2	−8.8 ± 6.2	−10.6, −7.0	0.046 *

*p* values were calculated using the unpaired *t*-test or Fisher’s exact test. * *p* < 0.05. SD, standard deviation; CI, confidence interval; PG, primary open-angle glaucoma; EG, exfoliation glaucoma; BCVA, best-corrected visual acuity; LogMAR, logarithm of minimum angle of resolution; IOP, intraocular pressure; MS, medication score; SERE, spherical equivalent refractive error; D, diopter; MD, mean deviation; dB, decibel.

**Table 2:** IOP at admission and discharge.

Parameters	Glaucoma Medication (+) n = 414		Glaucoma Medication (−) n = 73		*p* Value ^b^
	N or Mean ± SD	% or 95% CI Range	N or Mean ± SD	% or 95% CI Range	
**Non-surgical eye**					
IOP at admission, mmHg	14.8 ± 5.5	14.3, 15.4	13.9 ± 6.0	12.5, 15.2	0.17
IOP at discharge, mmHg	13.2 ± 5.8	12.6, 13.8	13.3 ± 6.9	11.7, 14.9	0.89
*p* value ^a^	<0.0001 *		0.33		
ΔIOP, mmHg	−1.6 ± 4.8	−2.1, −1.2	−0.6 ± 4.8	−1.7, 0.57	0.080
%ΔIOP, %	−7.5 ± 28.7	−10.3, −4.7	−1.1 ± 31.3	−8.4, 6.2	0.084
**Surgical eye**					
IOP at admission, mmHg	18.3 ± 8.7	17.5, 19.2	19.4 ± 9.3	17.2, 21.5	0.36
IOP at discharge, mmHg	10.1 ± 8.0	9.3, 10.9	9.3 ± 6.2	7.8, 10.7	0.43
*p* value ^a^	<0.0001 *		<0.0001 *		
ΔIOP, mmHg	−8.3 ± 11.5	−9.4, −7.1	−10.1 ± 11.5	−12.7, −7.4	0.22
%ΔIOP, %	−36.0 ± 54.0	−41.1, −30.7	−40.1 ± 50.9	−52.0, −28.3	0.53

*p* values were calculated using the paired *t*-test (^a^) and the unpaired *t*-test (^b^). * *p* < 0.05. SD, standard deviation; CI, confidence interval; IOP, intraocular pressure; ΔIOP, IOP at discharge minus IOP at admission; %ΔIOP, ΔIOP divided by IOP at admission.

**Table 3:** MS at admission and discharge.

Parameters	Glaucoma Medication (+) n = 414		Glaucoma Medication (−) n = 73		*p* Value ^b^
	N or Mean ± SD	% or 95% CI Range	N or Mean ± SD	% or 95% CI Range	
**Non-surgical eye**					
MS at admission	3.1 ± 1.2	3.0, 3.2	0 ± 0	0, 0	<0.0001 *
MS at discharge	2.3 ± 1.3	2.2, 2.4	0.5 ± 1.0	0.3, 0.7	<0.0001 *
*p* value ^a^	<0.0001 *		<0.0001 *		
ΔMS	−0.9 ± 1.3	−1.0, −0.7	0.5 ± 1.0	0.3, 0.7	<0.0001 *
%ΔMS, %	−22.5 ± 40.3	−26.4, −18.6	-	-	-
**Surgical eye**					
MS at admission	3.3 ± 1.2	3.2, 3.4	1.5 ± 1.6	1.2, 1.9	<0.0001 *
MS at discharge	1.1 ± 1.2	1.0, 1.3	0.9 ± 1.2	0.6, 1.1	0.072
*p* value ^a^	<0.0001 *		0.001 *		
ΔMS	−2.2 ± 1.7	−2.3, −2.0	−.7 ± 1.8	−1.1, −0.3	<0.0001 *
%ΔMS, %	−61.0 ± 43.7	−65.3, −56.8	−64.2 ± 41.9	−77.6, −50.8	0.64

*p* values were calculated using the paired *t*-test (^a^) and the unpaired *t*-test (^b^). * *p* < 0.05. SD, standard deviation; CI, confidence interval; ΔMS, medication score at discharge minus medication score at admission; %ΔMS, ΔMS divided by medication score at admission.

**Table 4:** Possible associations between ΔIOP and various parameters analyzed by a multiple regression model.

Parameter	Glaucoma Medication (+) n = 414			Glaucoma Medication (−) n = 73		
	Estimate	95% CI	*p* Value	Estimate	95% CI	*p* Value
Age, years	−0.04	−0.09, 0.002	0.059	−0.07	−0.2, 0.03	0.16
Sex, female/male	−0.03	−0.5, 0.4	0.91	0.4	−0.4, 1.3	0.29
Mini-Cog score, ≤2/≥3	−0.4	−2.4, 1.6	0.69	−2.3	−5.5, 0.8	0.14
Glaucoma type						
EG/PG	−0.5	−1.3, 0.4	0.32	0.6	−0.8, 2.1	0.41
other/PG	0.4	−0.3, 1.2	0.27	−1.3	−2.6, −0.04	0.044 *
BCVA, LogMAR	−1.0	−2.6, 0.7	0.26	0.06	−2.5, 2.7	0.96
SERE, D	−0.007	−0.2, 0.14	0.92	−0.09	−0.4, 0.2	0.51
IOP at admission, mmHg	−0.5	−0.6, −0.4	<0.0001 *	−0.6	−0.8, −0.4	<0.0001 *
MS at admission, med	0.08	−0.3, 0.4	0.65	-	-	-
MD, dB	−0.005	−0.07, 0.06	0.89	−0.05	−0.2, 0.1	0.52
Length of hospital stay, days	0.3	0.03, 0.6	0.029 *	−0.5	−1.1, 0.2	0.15

*p* values were calculated using a multiple regression model. * *p* < 0.05. PG, primary open-angle glaucoma; EG, exfoliation glaucoma; BCVA, best-corrected visual acuity; LogMAR, logarithm of minimum angle of resolution; SERE, spherical equivalent refractive error; D, diopter; IOP, intraocular pressure; MS, medication score; MD, mean deviation; dB, decibel.

**Table 5:** Possible associations between %ΔIOP and various parameters analyzed by a multiple regression model.

Parameter	Glaucoma Medication (+) n = 414			Glaucoma Medication (−) n = 73		
	Estimate	95% CI	*p* Value	Estimate	95% CI	*p* Value
Age, years	−0.3	−0.6, −0.03	0.028 *	−0.4	−1.2, 0.3	0.25
Sex, female/male	−0.8	−3.6, 1.9	0.56	3.8	−3.0, 10.5	0.27
Mini-Cog score, ≤2/≥3	−3.9	−16.8, 8.9	0.55	−18.5	−43.6, 6.7	0.15
Glaucoma type						
EG/PG	−3.9	−9.5, 1.8	0.18	3	−8.8, 14.7	0.61
other/PG	2.7	−2.2, 7.5	0.28	−11.1	−21.6, −0.7	0.037 *
BCVA, LogMAR	−3.1	−13.9, 7.7	0.57	−1.4	−22.4, 19.7	0.90
SERE, D	0.4	−0.5, 1.3	0.40	−0.5	−2.8, 1.8	0.65
IOP at admission, mmHg	−2.5	−3.0, −1.9	<0.0001 *	−3.4	−5.0, −1.8	<0.0001 *
MS at admission, med	0.1	−2.1, 2.4	0.90	-	-	-
MD, dB	0.1	−0.3, 0.5	0.64	−0.9	−2.2, 0.4	0.17
Length of hospital stay, days	2.1	0.4, 3.8	0.014 *	−5.4	−10.7, −0.1	0.045 *

*p* values were calculated using a multiple regression model. * *p* < 0.05. PG, primary open-angle glaucoma; EG, exfoliation glaucoma; BCVA, best-corrected visual acuity; LogMAR, logarithm of minimum angle of resolution; SERE, spherical equivalent refractive error; D, diopter; IOP, intraocular pressure; MS, medication score; MD, mean deviation; dB, decibel.

**Table 6:** Possible associations between ΔMS and various parameters analyzed by a multiple regression model.

Parameter	Glaucoma Medication (+) n = 414			Glaucoma Medication (−) n = 73		
	Estimate	95% CI	*p* Value	Estimate	95% CI	*p* Value
Age, years	−0.02	−0.03, −0.007	0.0026 *	0.01	−0.03, 0.05	0.57
Sex, female/male	−0.009	−0.1, 0.1	0.89	0.002	−0.3, 0.3	0.99
Mini-Cog score, ≤2/≥3	0.05	−0.5, 0.6	0.87	−0.2	−1.4, 1.0	0.75
Glaucoma type						
EG/PG	0.03	−0.2, 0.3	0.82	−0.08	−0.6, 0.5	0.76
other/PG	−0.06	−0.3, 0.2	0.57	−0.03	−0.5, 0.5	0.90
BCVA, LogMAR	−0.4	−0.9, 0.08	0.10	−0.4	−1.4, 0.6	0.38
SERE, D	0.01	−0.03, 0.05	0.60	−0.05	−0.2, 0.06	0.35
IOP at admission, mmHg	0.003	−0.02, −0.03	0.84	0.06	−0.01, 0.1	0.11
MS at admission, med	−0.6	−0.7, −0.5	<0.0001 *	-	-	-
MD, dB	−0.03	−0.05, −0.009	0.005 *	0.002	−0.06, 0.06	0.93
Length of hospital stay, days	−0.06	−0.1, 0.01	0.10	0.2	−0.08, −0.4	0.19

*p* values were calculated using a multiple regression model. * *p* < 0.05. PG, primary open-angle glaucoma; EG, exfoliation glaucoma; BCVA, best-corrected visual acuity; LogMAR, logarithm of minimum angle of resolution; SERE, spherical equivalent refractive error; D, diopter; IOP, intraocular pressure; MS, medication score; MD, mean deviation; dB, decibel.

**Table 7:** Possible associations between %ΔMS and various parameters analyzed by a multiple regression model.

Parameter	Glaucoma Medication (+) n = 414		
	Estimate	95% CI	*p* Value
Age, years	−0.7	−1.1, −0.3	0.001 *
Sex, female/male	−0.2	−4.4, 4.0	0.92
Mini-Cog score, ≤2/≥3	6.2	−13.4, 25.7	0.54
Glaucoma type			
EG/PG	1.1	−7.5, 9.7	0.80
other/PG	−2.7	−10.0, 4.6	0.47
BCVA, LogMAR	−13.0	−29.3, 3.4	0.12
SERE, D	1.1	−0.3, 2.5	0.12
IOP at admission, mmHg	0.2	−0.7, −1.1	0.66
MS at admission, med	−11.6	−15.1, −8.2	<0.0001 *
MD, dB	−0.6	−1.3, 0.05	0.069
Length of hospital stay, days	−1.9	−4.4, 0.7	0.15

*p* values were calculated using a multiple regression model. * *p* < 0.05. PG, primary open-angle glaucoma; EG, exfoliation glaucoma; BCVA, best-corrected visual acuity; LogMAR, logarithm of minimum angle of resolution; SERE, spherical equivalent refractive error; D, diopter; IOP, intraocular pressure; MS, medication score; MD, mean deviation; dB, decibel.

## Data Availability

Data are fully available upon reasonable request to the corresponding author.
